# Use of Sponge-Foam Inserts in Compression Bandaging of Non-Healing Venous Leg Ulcers

**DOI:** 10.3400/avd.oa.20-00159

**Published:** 2021-03-25

**Authors:** Rica Tanaka, Hideaki Inoue, Takeru Ishikawa, Yuichi Ichikawa, Rumiko Sato, Azusa Shimizu, Hiroshi Mizuno

**Affiliations:** 1 Department of Plastic and Reconstructive Surgery, Juntendo University School of Medicine, Sapporo, Hokkaido, Japan; 2 Division of Regenerative Therapy, Juntendo University Graduate School of Medicine, Tokyo, Japan; 3 ALCARE Co., Ltd., Tokyo, Japan

**Keywords:** compression therapy, sponge-foam inserts, venous leg ulcers

## Abstract

**Objective:** Venous leg ulcers (VLUs) caused by chronic venous insufficiency are difficult to treat. Outcomes after compression therapy and the current standard of care often used in conjunction with other options vary widely. We examined the effects of foam inserts on sub-bandage pressures in patients with VLUs and compared use of foam inserts in elastic and inelastic compression bandaging.

**Methods:** Six patients (≥20 years old) with VLUs and skin perfusion pressure >40 mmHg were included. Each patient underwent weekly treatment regimens of debridement, dressing changes, and dual sponge-insert application followed by elastic (n=3) or inelastic (n=3) compression bandaging. The median resting sub-bandage pressures of the ulcer beds, wound sizes, and healing percentages were recorded. Wound beds were biopsied before and after treatment for histological assessment. Nine healthy volunteers served as controls during preliminary testing.

**Results:** With proper sub-bandage pressures (>35 mmHg), the average healing time was 88.0±66 days, which was shorter than anticipated (i.e., ≥6 months). Combining large and local sponge-foam inserts increased sub-bandage pressures regardless of the compression bandage selected, with marked improvements seen in deeper wounds.

**Conclusion:** Layering one or two sponge-foam inserts beneath compression bandages facilitates uniform and optimal wound-bed pressure, which accelerates the healing of VLUs.

## Introduction

Venous leg ulcers (VLUs) are an insidious consequence of chronic venous insufficiency and are a healthcare burden, affecting approximately 1.2 million adults in the United States alone.^1)^ The key contributor to the development of VLUs is vascular hyperpermeability (due to prolonged venous pressure elevation and microangiopathy),^2)^ causing localized inflammation and eventual ulceration through the seepage of blood cells, macromolecules, and fluids.^3,4)^ Such inherently poor tissue conditions similarly deter wound healing, making treatment particularly difficult.^5,6)^

The current standard of care for VLU is compression therapy, specifically wrapping of the calf muscle in a bandage to passively raise interstitial pressure, promote venous return, and end the cycle of deleterious events.^7–9)^ Although it is proven to accelerate healing, success rates of this approach vary considerably in published reports (40%–95%),^5,8,10)^ with a high likelihood of recurrence (>72%).^11,12)^ Furthermore, sustained^13–15)^ and sufficiently intense (34–55 mmHg) sub-bandage pressure levels are essential for optimal results.^16–20)^

A wide variety of compression bandages (elastic and inelastic) are currently available, with some having built-in tension guides for self-monitoring of bandage pressures by patients. However, exerting ideal sub-bandage pressure is often precarious in deep ulcers (>2 cm depth)^5)^ or in ulcers situated over or near bony prominences (such as malleoli).^21)^ To ensure desired pressure levels, kidney-shaped foam fillers or gauze pads are often placed between the ulcer and the bandage.^21)^ We had adopted a strategy of layering one or two sponge-foam inserts beneath the compression bandages to facilitate uniform and optimal wound-bed pressure.

Herein, we report the effects of foam inserts on sub-bandage pressure, comparing the results in instances of elastic and inelastic compression bandaging. The outcomes of this technique are also highlighted in two patient presentations.

## Methods

Initially, it was necessary to select a sufficiently compressive inelastic bandage to test and determine a sponge-foam thickness optimal for this setting. Nine healthy volunteers were recruited for this study. Our bandaging technique was then tested in six patients (≥20 years old) with non-healing VLUs. The cases were reported to the institutional review board (IRB) (IRB approval no. JHS 17–0049) for publication approval. All patients provided informed consent to receive treatment.

### Preliminary selection of inelastic bandage and foam-insert thickness

Inelastic bandages without or with tension guides are currently available on the market (Elascot/Elascot-Tension Guide [Elascot-TG]; Alcare Co., Ltd., Tokyo, Japan). For this reason, we first investigated their capacity to reach a pressure of 30 mmHg in nine healthy volunteers. With each subject supine, median resting sub-bandage pressure was determined using a pressure transducer (MST MK III; Salzmann MEDICO, St Gallen, Switzerland). Desired sub-bandage pressures were achieved using Elascot-TG bandages (31±8 mmHg; Elascot, 25±8 mmHg), which were the bandages of choice.

Sub-bandage pressures were measured after placing 5-mm- or 10-mm-thick reticulated foam sponge inserts (Everlight SF HZ-80; Bridgestone Corp., Tokyo, Japan) on the calves of these healthy volunteers and applying compression bandages. The 10 mm inserts generated sub-bandage pressures that were 1.7-fold greater than those of 5 mm inserts and were thus selected for the study.

### Healing outcomes in patients with VLUs

The skin perfusion pressure (SPP) in each of the six participants was >40 mmHg. All subjects were candidates for compression therapy and maintenance debridement after screening with venous duplex imaging and magnetic resonance venography. None of the patients had deep vein thrombosis. The major reason for the wound was lower limb motor dysfunction. None of the patients were indicated to receive varicose surgery for wound treatment by a vascular surgeon. Therefore, no lower limb vein regurgitation was observed in the patients.

**Figure 1:**
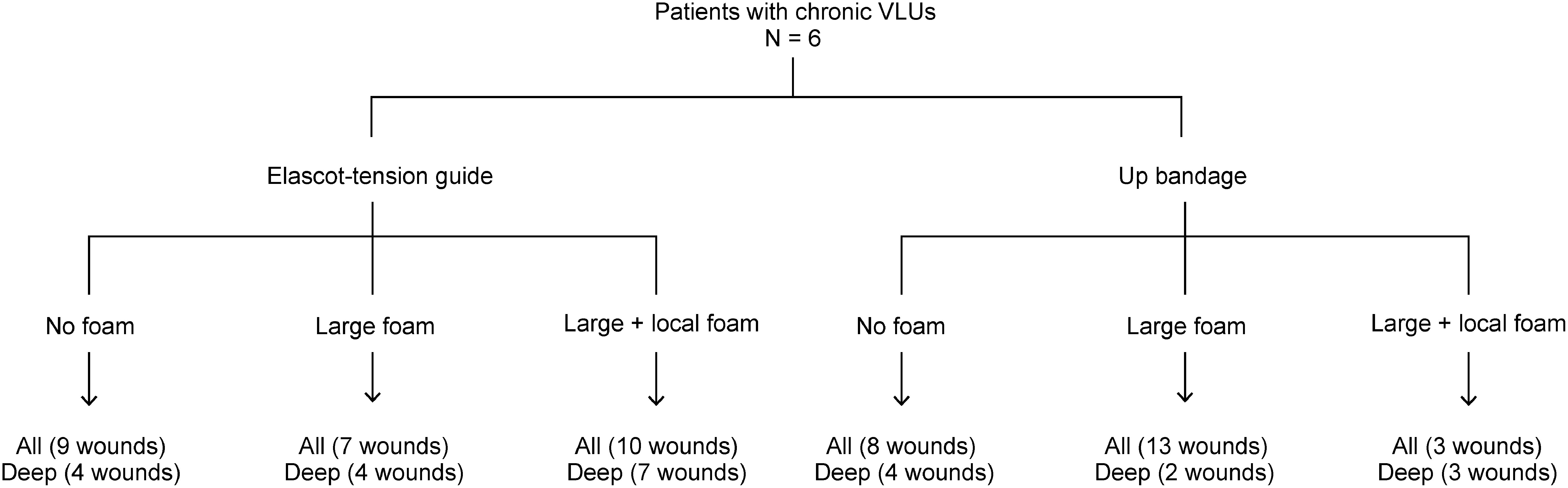
Fig. 1 Schematic of the study design.

**Figure 2:**
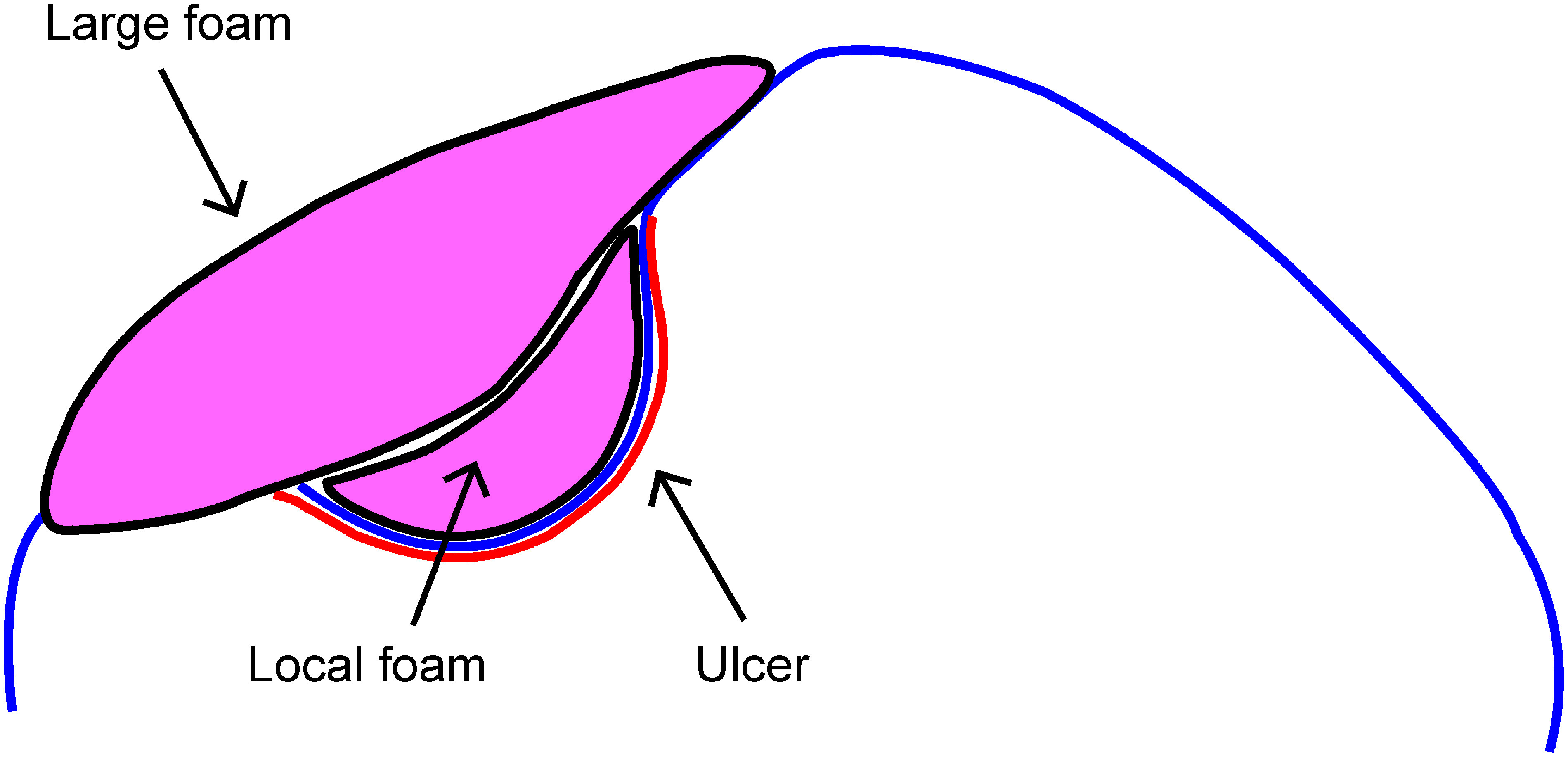
Fig. 2 Placement of large and local sub-bandage sponge-foam inserts.

The treatment regimen consisted of weekly wound curettage (mechanical debridement) and application of a primary dressing (SI-AID; Alcare Co., Ltd.), changed daily by the patients. A small local foam insert, cut to size for each ulcer, was placed atop the dressing and directly over the wound; thereafter, a second and larger foam insert was layered onto the first. Following this, an elastic compression bandage (up bandage; Kawamoto Corp., Osaka, Japan) or an inelastic tension-guided bandage (Elascot-TG; Alcare Co., Ltd.) was applied at the extremities, assigning three patients to each arm of treatment (**Figs. 1** and **2**).

The median resting sub-bandage pressures exerted over the ulcer beds were measured as previously described.^22)^ In addition, all VLUs were photographed once weekly, and wound sizes were measured by an independent plastic surgeon blinded to the study group. Wound healing percentages were determined from the wound size measurements.

### Statistical analysis

To compare the compression groups, the paired sample t-test and one-way ANOVA were applied. All computations were performed using standard software (GraphPad Prism v 6.0 for Windows; GraphPad Software Inc., San Diego, CA, USA), with statistical significance set at p<0.05.

## Results

### Shallow ulcers

**Figure 3:**
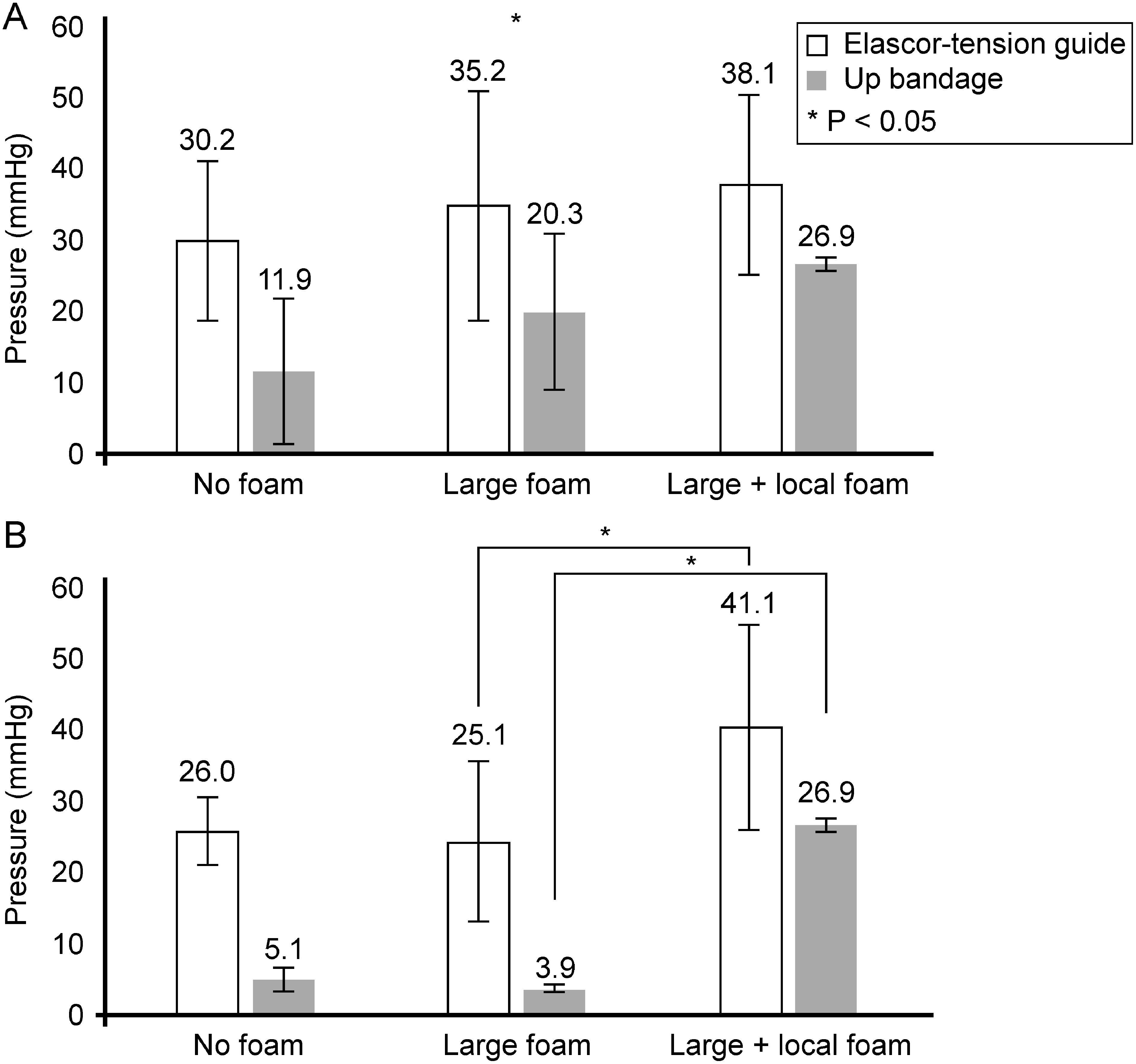
Fig. 3 Sub-bandage pressure exerted on affected sites using inelastic tension-guided bandages (Elascot-TG; n=3) or elastic bandages (up bandage; n=3), without or with sponge inserts (large and local foams) for (**A**) shallow ulcers and (**B**) deep ulcers.

Sub-bandage pressure was significantly higher in the inelastic tension-guided bandages than in the elastic bandages. In the absence of foam inserts, sub-bandage pressures were suboptimal in both groups. The larger foam insert alone increased sub-bandage pressure in both groups, although not to clinically desired levels. The addition of the smaller local foam insert resulted in a further increase of sub-bandage pressures (inelastic tension-guided bandage: 38.1±12.5 mmHg; elastic bandage: 26.9±1.0 mmHg) (**Fig. 3A**).

### Deep ulcers

Observed sub-bandage pressure amounts were less in deep (vs. shallow) ulcers for both test groups (elastic vs. inelastic tension-guided bandages), particularly if no or only one foam insert was used. As with shallow ulcers, inelastic tension-guided bandaging of deep ulcers led to higher sub-bandage pressure amounts than those due to elastic bandaging. Unlike shallow ulcers, however, use of only the large foam insert did not increase sub-bandage pressure in either group. By contrast, the addition of smaller local foam inserts significantly increased sub-bandage pressure in both groups (inelastic tension-guided bandage: 41.1±14.0 mmHg; elastic bandage: 26.9±1.0 mmHg) (**Fig. 3B**).

Wound healing percentages were estimated at 83%±2% in the six study patients during the treatment period of 88.0±66 days.

### Patient #1

**Figure 4:**
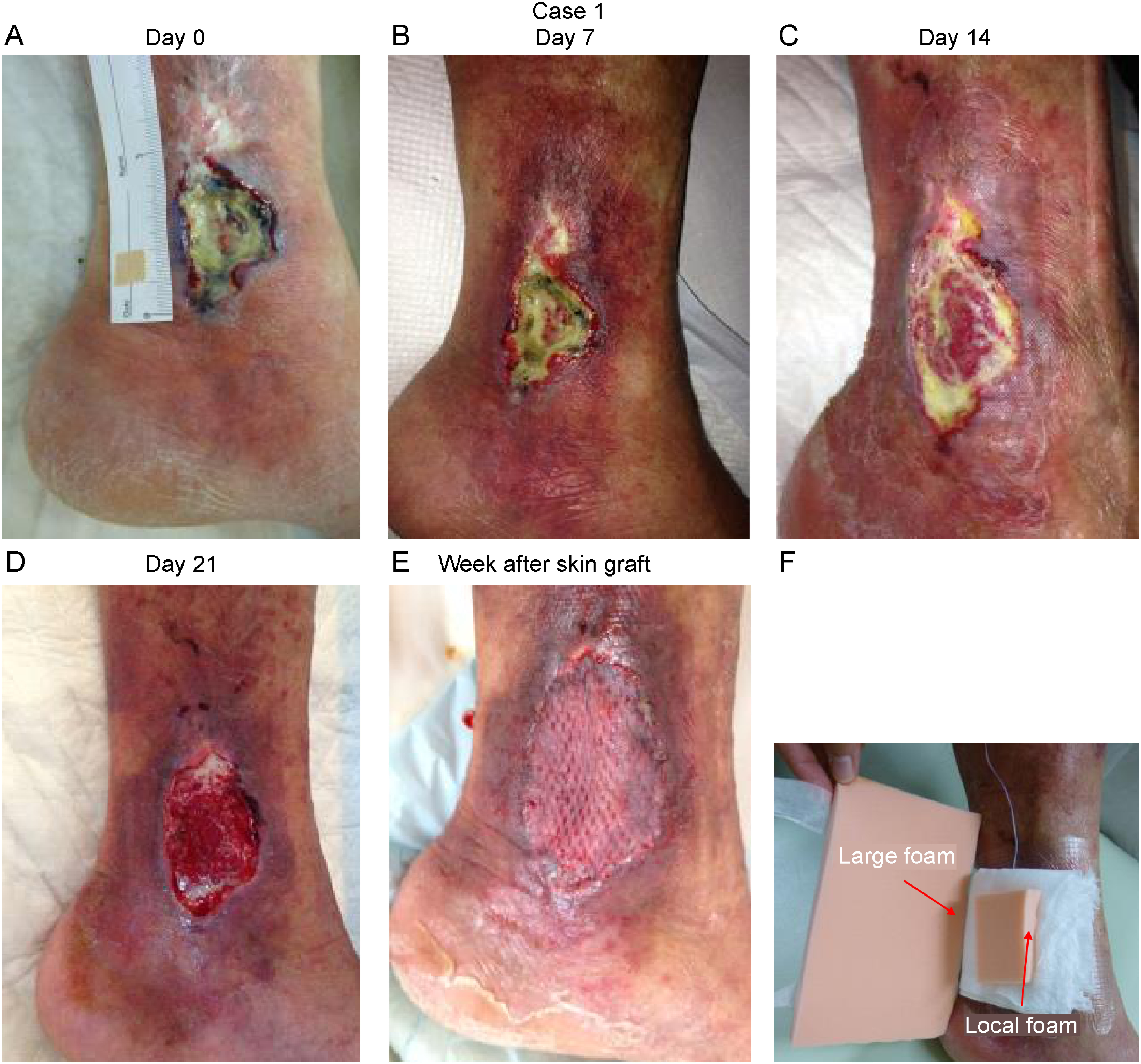
Fig. 4 Patient #1: Weekly photos of the ulcer of the right ankle (**A**–**D**) and 1 week after skin graft (**E**). Note the progressive reduction in leg edema and revitalization of tissues that promote wound healing. Dual-layer sponge-foam inserts (**F**) are illustrated on the right.

A 66-year-old man with a history of prolonged standing and 13 years of compression therapy for VLU presented with a chronic ulcer (5 cm×2.5 cm) on the right ankle. SPP and dorsal ankle-brachial index (ABI) were normal. Application of dual foam inserts increased sub-bandage pressure from 4 mmHg to 35 mmHg. The wound was debrided; foam inserts were applied, and an inelastic bandage was used for compression therapy. This resulted in significant improvement of the ulcer and reduction of leg edema, allowing split-thickness skin grafting after 3 weeks. Wound healing was complete 2 weeks after grafting (**Fig. 4**). The patient continued the compression therapy and remained ulcer-free for >18 months.

### Patient #2

**Figure 5:**
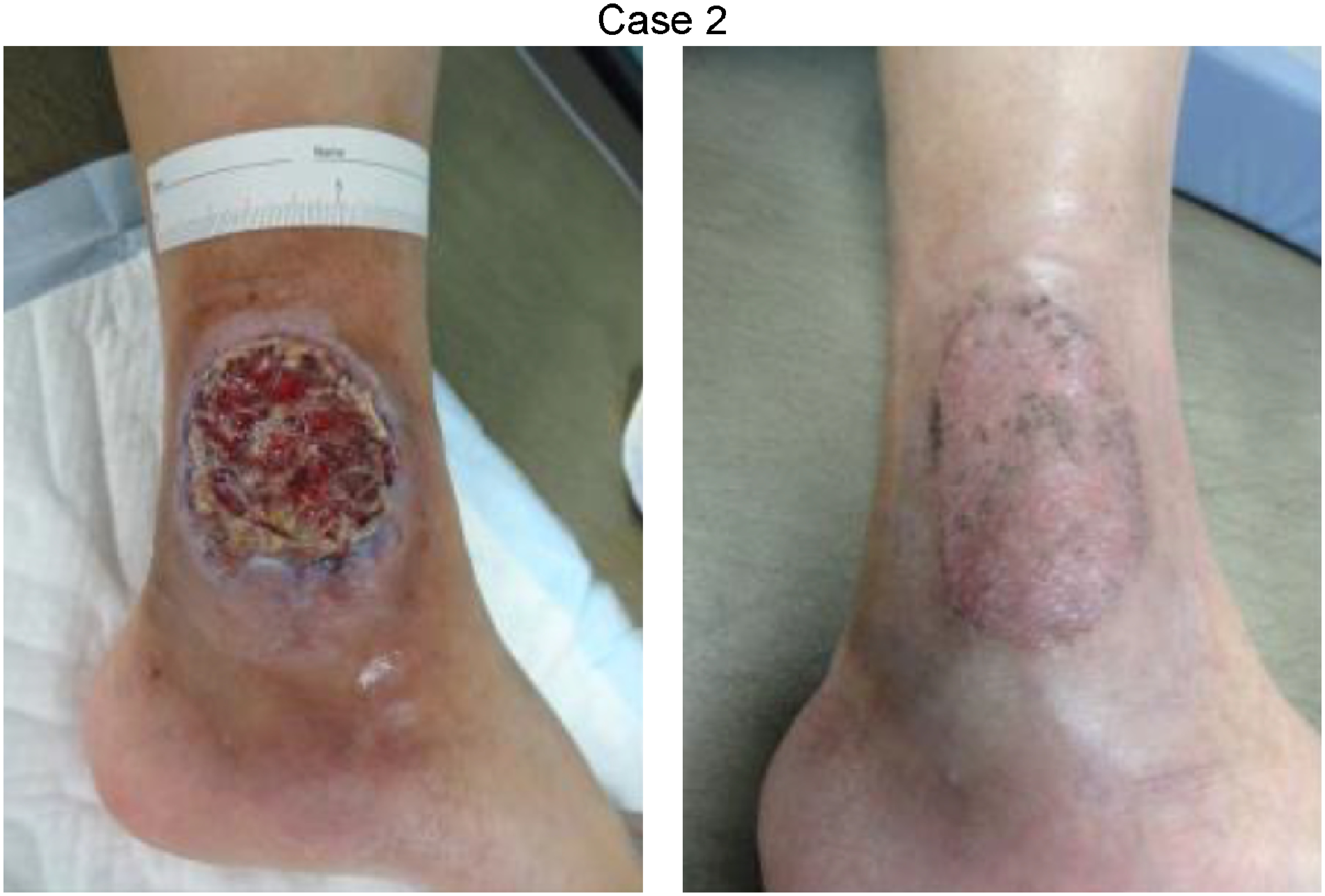
Fig. 5 Patient #2: A large, indurated, and hyper-pigmented ulcer of the left ankle (left) and the well-healed grafted site (right), with no signs of imminent recurrence.

A 58-year-old woman with a sedentary occupation but with no specific medical issues presented with a chronic ulcer (5.6 cm×5 cm) on the left ankle. Six months before ulcer development, the patient had undergone laser therapy, and 6 months prior to visiting our hospital, the wound had failed to heal despite compression therapy. The patient’s SPP and dorsal ABI were normal. The application of dual foam inserts increased sub-bandage pressure from 1.8 mmHg to 25.3 mmHg. Upon wound debridement, the use of foam inserts and an inelastic compression bandage visibly improved the ulcer and reduced leg edema. A split-thickness skin graft was performed 8 weeks later, with complete wound healing 2 weeks after grafting (**Fig. 5**). The patient continued compression therapy and remained ulcer-free after >12 months.

## Discussion

VLUs are difficult to cure, and their treatment can be costly, requiring significant resources. Currently, the standard of care for patients with VLUs is compression therapy, with various studies indicating that healing can be facilitated through sub-bandage pressure exertion. On average, the sub-bandage pressure sufficient for healing of VLUs ranges from 35–45 mmHg.^23)^ An earlier study also confirms that dual- or multi-component compression systems yield higher healing rates for VLUs than those due to single-component compression systems.^5)^ Nonetheless, it is evident that sub-bandage pressure application has merit in the context of large or anatomically constrained ulcers that are refractory to standard compression therapy.^5,24)^

Large VLUs typically arise on or near the ankle, where applying sub-bandage pressure is problematic. Without adequate sub-bandage pressure, fluid in adjacent tissues is forced into the wound itself by compression, causing ulcers to worsen. Given that standard compression therapy is not universally successful, it must be customized to ensure sufficient direct wound pressure. Thus, our strategy of applying sponge inserts beneath the bandage and directly over wounds was conceived.

In the present study, we enrolled six patients with VLUs. All subjects received both local sponge inserts and larger sponge-foam pads, each covered by elastic (n=3) or inelastic tension-guided (n=3) bandages. Although VLUs are otherwise slow to heal, often requiring >6 months to resolve (39%–50%), the average healing time in our patients was 88.0±66 days, which was shorter than expected. Thus, we showed that exerting proper sub-bandage pressure (>35 mmHg) on non-healing VLUs using sponge-foam inserts is a viable approach. To the best of our knowledge, this is the first report of accelerated wound healing through such a technique.

We also investigated elastic and inelastic compression bandages as variables in this process, discovering that regardless of bandage type, generating sufficient sub-bandage pressure is difficult without directly applying sponge-foam inserts to wounds. Initially, an insert that exceeded the size of the wound was utilized. We tried a smaller (local) insert cut precisely to the wound size as a base layer. The combined use of large and local sponge-foam inserts incrementally increased sub-bandage pressure acceptably for both groups.

The benefit conferred by local sponge-foam inserts was particularly visible when used on persistent deeper wounds. The locally placed sponge-foam compensated for surface depressions and irregularities, creating a circumferentially smooth surface to layer the large sponge. The principles entailed are embodied in Laplace’s law,^25)^ which stipulates that the bandage size must be in direct proportion to the radius of the leg to ensure sufficient sub-bandage pressure. In addition, a depressed wound lacking sufficient compression will worsen due to engorgement of fluid influx from nearby tissues. Similarly, the shape of the leg (especially close to the ankle) is another factor that can worsen the condition based on Laplace’s law. These findings indicate that applying dual sponge-foam inserts confers sufficient sub-bandage pressure, even if only a single-layer compression bandage is used.

### Limitations

A major limitation of our study was the lack of a control group in assessing the efficacy of these sponge-foam inserts. However, we believe that our reported methodology and outcomes were the main contributors to the observed accelerated healing of ulcers due to higher sub-bandage pressure in the present study.

## Conclusion

Although multi-layer compression systems have proved effective, our experience indicates that patients may abandon treatment if the process is too laborious. However, we have clearly demonstrated that single-layer tension-guided compression bandages produce sufficient sub-bandage pressure if sponge-foam inserts are applied. Such inserts are versatile and straightforward adjuncts to VLU treatment and are easily implemented in the patients’ routine daily dressing changes. The regulatory environment virtually excludes home nursing services, particularly in Japan; thus, most patients perform dressing changes on their own. Any common compression regimen may be tailored in this manner to accelerate wound healing with fewer associated physical or economic burdens.
